# Patterns of Physical Activity and Depressive Symptoms Among Korean Adults: A Descriptive Cross-Sectional Analysis of the 2023 Korea Community Health Survey

**DOI:** 10.3390/healthcare13243221

**Published:** 2025-12-09

**Authors:** Ah-Yoon Kim, Sang-A Nam, Su-Yeon Roh, Geun-Kook Kim

**Affiliations:** 1 Brain Science Research Institute, Seoul University of Buddhism, Seoul 08559, Republic of Korea; kimahyoon@gmail.com; 2 Department of Health Science, Gachon University Graduate School, Incheon 21936, Republic of Korea; yongyong1998@gachon.ac.kr; 3 Department of Exercise Rehabilitation, Gachon University, Incheon 21936, Republic of Korea; 4 Department of Sports Rehabilitation, Jaeneung University, Incheon 22573, Republic of Korea

**Keywords:** physical activity, depressive symptoms, descriptive epidemiology, population health, South Korea, COVID-19

## Abstract

**Background/Objectives:** Depression has increased substantially in Korea following the COVID-19 pandemic, with prevalence reaching 7.3% in 2023, the highest level in a decade, raising urgent concerns about widening mental health disparities. Although physical activity (PA) is associated with reduced depressive symptoms, nationally representative post-pandemic evidence from Korean adults remains limited. This study descriptively examined patterns of PA participation and depressive symptoms across key sociodemographic groups using 2023 Korea Community Health Survey (KCHS) data. **Methods:** We analyzed cross-sectional data from 228,249 adults aged ≥19 years in the 2023 KCHS. Depressive symptoms were measured using nine PHQ-9 items (1–4 on Likert scale). PA was assessed as the number of days per week (0–7) of moderate (MPA) and vigorous (VPA) physical activity according to KCHS operational definitions. All analyses incorporated complex survey features (strata, clusters, weights). Group differences were examined using design-corrected *t*-tests and ANOVA. **Results:** Women, adults aged 60 years or older, bereaved individuals, and those with lower educational attainment reported higher depressive symptom levels (*p* < 0.001). PA participation was higher among men, younger adults, married individuals, and those with higher education. Depressive symptom scores decreased with increasing PA frequency, with the lowest levels observed among adults active 5–7 days per week. Although mean differences were modest (0.02–0.12 points on the four-point scale; η^2^ < 0.06), these steady population-level gradients provide meaningful baseline information for understanding post-pandemic mental health patterns in Korea. **Conclusions:** Although individual-level differences were small (η^2^ < 0.06), the population-level gradients are important for monitoring mental health disparities in post-pandemic Korea. Women, older adults, bereaved individuals, and lower-education groups represent key high-burden populations. Future studies should employ longitudinal designs, objective PA measures, and confounder-adjusted models to clarify mechanisms and directionality, and evaluate the effectiveness of community-based PA interventions.

## 1. Introduction

Depression is a leading cause of disability worldwide, affecting more than 280 million people [1]. In Korea, the prevalence of depressive symptoms reached 7.3% in 2023, the highest level in the past decade [2]. These trends highlight the need for updated population-based data to understand mental health patterns in the post-pandemic context.

Physical activity (PA) is widely recognized as a modifiable behavior associated with improved mental health. Meta-analyses consistently report inverse associations between PA and both elevated depressive symptoms and major depressive disorder diagnosis [3,4]. A comprehensive 2022 synthesis of 218 cohort studies found that individuals engaging in even modest amounts of PA had significantly lower depression risk compared with inactive adults, showing a clear dose–response pattern [3]. However, most existing evidence is based on Western cohorts or pre-pandemic datasets. Limited research has examined how these associations may have shifted in Korea following COVID-19, a period marked by substantial changes in both PA behaviors and population mental health [5,6].

Prior Korean studies often relied on smaller or localized samples or lacked detailed subgroup analyses by sex, age, marital status, or education [7,8]. Given the social and behavioral changes that occurred during and after the pandemic, there is a critical need for nationally representative, post-pandemic population-level data to identify high-burden subgroups and describe current patterns.

The 2023 Korea Community Health Survey (KCHS) provides an opportunity to address this gap. As the most recent nationwide survey of over 220,000 adults, the KCHS allows for robust population-level estimation across key demographic strata [9].

Accordingly, this study aims to descriptively characterize patterns of PA and depressive symptoms among Korean adults using the 2023 KCHS. Specifically, we aim to (1) describe depressive symptom and PA patterns across sex, age, marital status, and educational groups; (2) identify population subgroups with higher depressive symptom burden and lower PA participation; and (3) examine the co-occurrence of these factors to inform hypotheses for future analytical studies.

## 2. Literature Review

### 2.1. Physical Activity and Depression: Evidence from Epidemiological Studies

A substantial body of epidemiological research demonstrates that regular physical activity (PA) is associated with lower levels of depressive symptoms and reduced risk of major depressive disorder. Meta-analyses of large-scale prospective cohort studies consistently report inverse associations across diverse adult populations [3,4]. A comprehensive 2022 synthesis of 218 cohort studies found that individuals engaging in even modest amounts of PA had significantly lower depression risk compared with inactive adults, showing a clear dose–response pattern [3]. Randomized controlled trials provide further support, demonstrating that structured aerobic exercise produces clinically meaningful reductions in depressive symptoms in both nonclinical and clinical populations [8,9].

Although these findings provide robust evidence for the protective association between PA and depression, most existing studies have been conducted in Western contexts. Differences in cultural norms, demographic structures, and PA patterns limit the applicability of these findings to Korean populations. Furthermore, COVID-19 disrupted daily movement routines and heightened psychological distress worldwide, underscoring the need for updated, population-level evidence reflecting post-pandemic conditions in Korea [10,11].

### 2.2. Biological, Psychological, and Social Mechanisms

Multiple biological, psychological, and social mechanisms explain why regular PA may protect against depression.

Biological mechanisms: PA induces acute and chronic changes within neural systems involved in mood regulation. One key pathway involves increases in brain derived neurotrophic factor (BDNF), which facilitates hippocampal neurogenesis and synaptic plasticity processes strongly linked to emotional regulation and cognitive function [12]. Exercise-related increases in BDNF have been consistently documented across controlled trials and meta-analyses. PA also influences stress physiology through regulation of the hypothalamic–pituitary–adrenal (HPA) axis. Stabilization of this system helps reduce stress reactivity and attenuates cortisol dysregulation commonly observed in depressive states [13]. In addition, PA enhances monoaminergic neurotransmission, including serotonin, dopamine, and norepinephrine neurotransmitter systems that play essential roles in mood regulation and are the primary targets of antidepressant medication [14]. Another relevant pathway involves reductions in inflammation, oxidative stress, and immune dysregulation; PA has been shown to downregulate proinflammatory signaling, which is increasingly recognized as a contributing factor to depression [13].

Psychological mechanisms: Regular PA promotes improvements in psychological resources that buffer against depressive symptoms. These include greater emotion regulation competence, enhanced self-efficacy, and reductions in maladaptive cognitive tendencies such as rumination [15]. Through these pathways, PA may facilitate more adaptive coping strategies, thereby reducing vulnerability to mood disturbances.

Social mechanisms: PA also provides important social benefits. Participation in walking groups, recreational sports, or community-based activity programs enhances social connectedness, reduces perceived isolation, and strengthens interpersonal support networks [16]. During and after the COVID-19 pandemic, when social distancing and routine disruptions intensified loneliness and emotional distress, these social pathways became even more salient for psychological well-being [17].

Together, these biological, psychological, and social processes offer a coherent explanation for the consistent inverse associations between PA and depressive symptoms observed in epidemiological research.

### 2.3. Evidence from Korean Studies and Post-Pandemic Trends

Korean research similarly indicates that adults with lower levels of PA tend to report higher depressive symptoms across age groups [6,18]. However, many earlier studies were conducted using pre-pandemic datasets such as KNHANES 2014–2019 or local community samples, limiting their ability to reflect contemporary behavioral and mental health conditions in Korea.

Recent data highlight notable post-pandemic shifts. National reports show that PA levels among Korean adults declined by approximately 12–15 percent compared with pre-pandemic years, while sedentary time increased substantially [18]. Concurrently, depressive symptoms increased nationwide, with prevalence rising from 5.8 percent in 2019 to 7.3 percent in 2023, the highest level recorded in the past decade [2].

Emerging evidence also points to widening disparities among sociodemographic groups. Women report substantially higher depressive symptom levels than men, and older adults have experienced pronounced increases in inactivity and psychological distress [18]. Individuals with lower socioeconomic status or disrupted social networks including bereaved adults also show elevated vulnerability to depressive symptoms [19]. These trends underscore the importance of examining PA and depression within the context of Korea’s post-pandemic social and behavioral environment.

Despite these observations, no published study has yet used the 2023 Korea Community Health Survey (KCHS) the most extensive nationally representative dataset collected after the pandemic to describe PA patterns and depressive symptoms across key demographic subgroups. This represents a meaningful gap in the current evidence base.

### 2.4. Research Gaps and Contribution of the Present Study

The existing literature reveals several important gaps.

First, nationally representative post-pandemic analyses of PA and depression in Korea remain limited, leaving uncertainties about how population-level patterns have shifted since COVID-19. Second, few studies have examined subgroup-specific differences across sex, age, marital status, and educational attainment, despite their relevance for understanding mental health disparities. Third, prior research has provided limited descriptive population-level data necessary for generating hypotheses to guide future analytical, mechanistic, or intervention studies. The present study addresses these gaps by utilizing the most recent nationally representative post-pandemic dataset (2023 KCHS), providing stratified descriptive estimates across key demographic strata, and identifying high-burden groups characterized by elevated depressive symptom levels and lower PA participation. These contributions offer an updated foundation for future epidemiologic investigations and intervention development in Korea.

## 3. Materials and Methods

### 3.1. Data Source and Sample Selection

This study did not involve the use of any laboratory equipment, materials, or devices, as it analyzed publicly available secondary data from the 2023 Korea Community Health Survey (KCHS). The KCHS, administered annually by the Korea Disease Control and Prevention Agency (KDCA), employs a multistage, stratified cluster sampling design to produce nationally representative estimates of Korean adults aged 19 years and older.

A total of 229,107 adults participated in the 2023 survey. Among them, 228,249 adults (99.6%) had complete information on both physical activity and PHQ-9 depressive symptom items and were included in the analytic sample. A total of 858 participants (0.4%) were excluded because of missing data on either variable. Data were collected via standardized, computer-assisted personal interviews conducted by trained field personnel.

### 3.2. Measures

#### 3.2.1. Depressive Symptoms

Depressive symptoms were measured using nine items derived from the Patient Health Questionnaire-9 (PHQ-9) [20,21]. Respondents rated each symptom on a four-point Likert scale (1 = never, 4 = almost every day) based on the previous two weeks. Items assessed diminished interest, depressed mood, sleep disturbance, fatigue, appetite changes, feelings of worthlessness, concentration difficulties, psychomotor changes, and suicidal ideation. Each item was analyzed individually to reflect symptom-specific variation rather than combined into a total score. In community surveys, the PHQ-9 is widely used as a screening instrument to assess symptom distribution rather than to diagnose major depressive disorder.

#### 3.2.2. Physical Activity (PA)

Physical activity (PA) was assessed according to 2023 KCHS operational definitions, which record the number of days per week (0–7 days) that respondents engaged in moderate physical activity (MPA) and vigorous physical activity (VPA). Because the KCHS measures PA frequency rather than duration or accumulated minutes, its PA metrics differ from duration-based guidelines such as those issued by the World Health Organization or the American College of Sports Medicine [22]. For this reason, PA variables were analyzed strictly as defined by the KCHS without applying external guideline thresholds.

#### 3.2.3. Demographic Variables

Demographic variables included sex, age group (19–39, 40–59, ≥60 years), marital status (married, unmarried, bereaved/divorced), and education level (middle school or less, high school, college or higher). These variables were used solely for descriptive stratification, consistent with the aims of this study.

### 3.3. Analytical Approach

#### 3.3.1. Complex Sampling Design

All analyses incorporated the full complex sampling structure of the 2023 KCHS [7,23], including stratification by region and administrative subdivision, cluster sampling units defined by community health center catchment areas, and sampling weights reflecting post-stratification to the 2023 Korean Census by sex, age, and region. Analyses were conducted using SPSS Complex Samples version 28.0 (IBM Corp., Armonk, NY, USA) [24], which provides design-based variance estimation appropriate for multistage survey data. Statistical testing employed the Rao and Scott adjusted F test for categorical variables [25] and design-adjusted *t*-tests for continuous variables [26], ensuring accurate variance estimation under complex sampling conditions.

#### 3.3.2. Descriptive Statistics and Group Comparisons

Weighted means and standard errors were calculated for each PHQ-9 item across demographic and PA-frequency groups. Group comparisons were conducted using design-adjusted *t*-tests and complex-samples ANOVA. When ANOVA results indicated statistical significance (*p* < 0.05), Bonferroni-adjusted pairwise comparisons were applied to control for Type I error associated with multiple testing.

#### 3.3.3. Effect Size Interpretation

Effect sizes (eta-squared, η^2^) were calculated for group comparisons and interpreted using conventional thresholds: small (<0.06), medium (0.06–0.14), and large (≥0.14) [27,28]. Because the PHQ-9 uses a restricted four-point scale and depressive symptoms in population-level data tend to show limited variability, small effect sizes were expected.

#### 3.3.4. Rationale for Descriptive Analysis and Confounder Considerations

This study was designed as a descriptive epidemiologic analysis aimed at characterizing population-level patterns rather than estimating causal associations. Consequently, no regression models or confounder adjustments were performed. Potential confounders such as chronic illness, socioeconomic status, social support, or health literacy were not included. These considerations are discussed as limitations and should be addressed in future longitudinal or confounder-adjusted studies. This analytical approach aligns with established descriptive epidemiologic principles and the study’s goals of providing updated population-based evidence for hypothesis generation.

## 4. Results

### 4.1. Sample Characteristics

Table 1 summarizes the weighted sociodemographic characteristics of the 228,249 adults included in the analysis. The sample comprised 54.0% women, and 46.6% were aged 60 years or older. Overall, 40.3% had completed college or higher education, and 63.5% were married.

### 4.2. Depressive Symptoms by Demographic Subgroups

**Figure 1:**
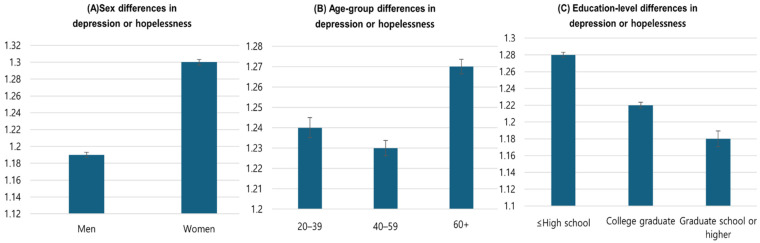
Sociodemographic subgroup differences in mean “depression or hopelessness” scores. (**A**) Sex, (**B**) age-group, and (**C**) education-level differences. Error bars represent 95% confidence intervals.

Table 2 presents weighted mean PHQ-9 item scores across demographic groups. Women reported significantly higher mean scores than men for all nine depressive symptom items (*p* < 0.001; η^2^ = 0.02–0.06). Depressive symptom levels increased significantly with age, with the highest values observed among adults aged ≥60 years compared to younger age groups (*p* < 0.001; η^2^ = 0.03–0.08). Bereaved or divorced individuals consistently reported significantly higher symptom levels than married adults (*p* < 0.001; η^2^ = 0.01–0.04). Participants with lower educational attainment (high school graduate or less) also reported significantly higher depressive symptom scores compared with college-educated adults (*p* < 0.001; η^2^ = 0.02–0.07). Figure 1 visualizes these sociodemographic disparities, highlighting significant differences by sex, age, and education level.

### 4.3. Physical Activity Patterns by Demographic Subgroups

Table 3 summarizes moderate physical activity (MPA) and vigorous physical activity (VPA) frequency by demographic characteristics. Men engaged in significantly higher physical activity than women, with mean differences of approximately 0.36 days per week for moderate-intensity activity and 0.45 days per week for vigorous-intensity activity (*p* < 0.001; η^2^ = 0.04–0.07). Physical activity participation decreased significantly with age, with the lowest levels observed among adults aged ≥ 60 years (*p* < 0.001; η^2^ = 0.05–0.09). Individuals with lower educational attainment reported substantially fewer days of MPA and VPA compared with college-educated adults (*p* < 0.001; η^2^ = 0.03–0.06).

### 4.4. Depressive Symptoms by Physical Activity Frequency

**Figure 2:**
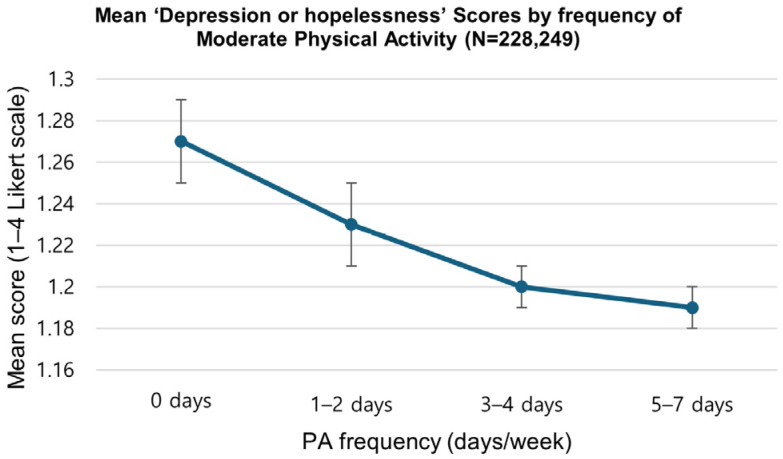
Pattern of mean PHQ-9 “depression or hopelessness” scores by moderate physical activity frequency (days/week). The figure shows an inverse dose–response pattern, with the lowest depressive symptoms at 5–7 days of activity per week. Error bars indicate standard errors.

Table 4 displays mean PHQ-9 item scores by weekly PA frequency (0, 1–2, 3–4, and 5–7 days). Across all items, individuals active 5–7 days per week showed the lowest depressive symptom scores (range: 1.12 to 1.34), while those reporting 0 days of activity showed the highest levels (range: 1.25 to 1.48). Mean differences across PA categories ranged from approximately 0.02 to 0.12 points on the four-point scale. All group comparisons were statistically significant (*p* < 0.001). As illustrated in Figure 2, a clear inverse dose–response relationship was observed, where depressive symptom scores progressively decreased as physical activity frequency increased.

## 5. Discussion

This descriptive epidemiological study examined patterns of physical activity and depressive symptoms among 228,249 Korean adults using nationally representative 2023 KCHS data. The key findings were that women, older adults, bereaved individuals, and those with lower educational attainment reported higher depressive symptoms, that these same groups showed lower participation in physical activity, and that higher physical activity frequency was consistently associated with lower depressive symptom scores in all demographic subgroups. Although effect sizes were small, the associations followed a stable pattern across the population.

### 5.1. Individual-Level Effect Sizes and Population-Level Significance

The small effect sizes observed in this study are expected in population-based monitoring research. The PHQ-9 uses a restricted four-point scale, limiting variability, and depressive symptoms in community samples tend to cluster within a relatively narrow range [29]. These methodological constraints reduce the magnitude of observable individual-level differences. However, even small differences can translate into meaningful population-level benefits. For example, a mean reduction of 0.05 points applied across Korea’s adult population of approximately 42 million could represent more than 2 million person-level symptom improvements. As highlighted in recent epidemiological perspectives, a relatively small shift in mean mental health scores often indicates a large shift in the number of cases crossing clinical thresholds at the tail of the distribution [30]. This illustrates how consistent population gradients can contribute to substantial aggregate gains and underscores the importance of population-level intervention strategies to address identified disparities, as supported by recent national baseline data [31].

### 5.2. Sex Differences: Mechanisms and Implications

Women consistently reported higher depressive symptoms and lower levels of physical activity than men, a pattern well documented in recent global analyses [32]. Multiple biological, psychosocial, and structural mechanisms contribute to these disparities. Biologically, hormonal fluctuations and neuroendocrine responses across the reproductive lifespan influence mood regulation and vulnerability to depression [33]. Furthermore, recent reviews highlight sex differences in stress susceptibility, driven by interactions between inflammatory, hormonal, and epigenomic mechanisms, as key contributors to the higher prevalence of depression in women [34]. Psychosocially and structurally, women experience distinct barriers including disproportionate caregiving responsibilities and cultural expectations that limit participation in sport or structured physical activity [35]. Post-pandemic data suggest these disparities may have widened, necessitating targeted interventions such as women-focused exercise groups and flexible digital activity programs.

### 5.3. Bereavement and Depressive Symptoms

Depressive symptoms were highest among bereaved adults, who also showed the lowest physical activity levels. This finding aligns with contemporary research linking spousal loss to prolonged grief disorder, systemic inflammation, and elevated mortality risk [36]. The mechanisms linking bereavement to increased depression and reduced physical activity are multifaceted. Bereaved individuals often experience a collapse of social regulation and support systems, which are critical buffers against stress. Conversely, physical activity may support adaptive adjustment by enhancing emotional regulation and facilitating gradual social reintegration. Recent evidence suggests that structured aerobic exercise can mitigate grief-related symptoms and improve psychological functioning in bereaved older adults [37].

### 5.4. Education as a Social Determinant

Adults with lower educational attainment reported higher depressive symptom levels and lower physical activity participation, a pattern reflecting broader social determinant mechanisms. Educational attainment is closely linked to health literacy the capacity to understand health information and make informed health decisions [38]. Lower education is associated with reduced understanding of depression and physical activity benefits, limited access to health-promoting resources, and greater economic constraints on health-related behaviors. This aligns with robust causal evidence published in *Science*, which demonstrates that adverse socioeconomic conditions, including lower education and poverty, directly increase exposure to chronic stressors while depleting psychological coping resources [39].

### 5.5. Dose–Response Patterns and Exercise Intensity

A graded inverse relationship between physical activity frequency and depressive symptoms was observed. Individuals active five to seven days per week reported the lowest symptom levels, while inactive adults reported the highest levels. Notably, moderate-intensity physical activity showed the clearest and most consistent dose–response pattern. This finding aligns with the WHO 2020 guidelines and recent mega-analyses suggesting that moderate-intensity activities which are inherently more sustainable and better tolerated provide optimal mental health benefits for the general population [40,41]. These patterns imply that promoting accessible, moderate-intensity community-based physical activities may be more effective than emphasizing high-intensity exercise.

### 5.6. Strengths and Limitations

Strengths include the large, nationally representative sample (N = 228,249), post-pandemic timing, comprehensive demographic stratification, appropriate application of complex survey weighting, and explicit effect-size reporting. These features enhance the robustness and public health relevance of the descriptive findings. Limitations include the cross-sectional design, which precludes causal inference and allows for potential reverse causality; reliance on self-reported physical activity and depressive symptoms; lack of detailed information on physical activity type, duration, or intensity beyond frequency; and unmeasured confounders including chronic illness, socioeconomic status (beyond education), social support, and health literacy. Additionally, PHQ-9 item-level analysis reflects symptom burden rather than clinical diagnosis of major depressive disorder.

### 5.7. Public Health and Policy Implications and Recommendations

The findings support a multilevel approach to physical activity-based depression prevention. This aligns with the WHO’s Global Action Plan on Physical Activity, which utilizes a systems-based ecological framework to promote active societies through upstream policy actions and community-wide initiatives [42,43].

At the universal level, national public health campaigns and community-wide infrastructure development should promote accessible moderate-intensity activities such as walking and recreational sports. At the targeted level, interventions must deliberately address the specific barriers identified for high-risk groups in this study. For women, policies should support safety-focused exercise environments and childcare services to enable participation. For older adults, age-adapted programs (e.g., Tai Chi, fall-prevention exercises) integrated into senior centers are recommended. For bereaved or divorced individuals, group-based programs offering peer support can help mitigate social isolation. Additionally, for populations with lower education levels, simplified public health messaging and accessible, low-cost community options should be prioritized to reduce health inequalities. Finally, at the clinical level, brief physical activity assessments and prescriptions should be systematically integrated into routine mental health services to ensure that physical activity is utilized as a core component of depression management.

### 5.8. Future Research Directions

Future studies should employ longitudinal or randomized experimental designs to establish temporal directionality and causal inference. Incorporation of objective physical activity measurement (e.g., accelerometers) is recommended to reduce recall bias, as recent large-scale studies have demonstrated their superior predictive value for health outcomes compared to self-reports [44]. Additional mechanistic research should investigate neurobiological pathways, such as the upregulation of Brain-Derived Neurotrophic Factor (BDNF) and anti-inflammatory cytokines [45], as well as psychological and social processes underpinning the antidepressant effects of exercise [46,47]. Studies specifically targeting high-burden groups identified in this analysis (women, older adults, bereaved persons, lower-education populations) may support development of more precisely tailored interventions.

## 6. Conclusions

This descriptive epidemiological study of 228,249 Korean adults revealed that depressive symptoms were consistently higher among women, older adults aged ≥ 60 years, bereaved individuals, and those with lower educational attainment. These same groups simultaneously demonstrated markedly lower physical activity participation. Across all demographic subgroups, a clear dose response pattern was observed: higher PA frequency was consistently associated with lower depressive symptom scores, with the lowest levels observed among individuals active 5–7 days per week [40,41]. Although individual-level effect sizes were small, the consistency of these population-level gradients across a large nationally representative sample highlights meaningful implications for public health monitoring and intervention development [30,31].

These findings support physical activity promotion as a feasible, evidence-based, and low-cost strategy for supporting mental health in community populations, as emphasized in recent global economic analyses [48]. The dose response analysis revealed that moderate-intensity PA demonstrated a clearer linear benefit trajectory than vigorous PA. This suggests that moderate-intensity activities which are more accessible, sustainable, and conductive to habit formation in community settings may offer a particularly effective approach for depression prevention across diverse populations [49].

The demographic disparities identified in this study highlight specific priority populations for targeted public health interventions, including women, older adults, bereaved individuals, and adults with lower educational attainment [32,35]. Tailored strategies addressing sex-specific, age-specific, and education-related barriers may enhance intervention effectiveness and reduce mental health disparities [42]. Future research should employ longitudinal and randomized experimental designs to establish causal directionality [44]. Incorporation of objective PA measurement (accelerometers) would reduce recall bias and enable characterization of activity patterns by intensity and type. Furthermore, future studies investigating neurobiological mechanisms (BDNF, inflammation) [45] and psychosocial pathways will clarify the mechanistic links between PA and mental health outcomes.

Overall, this descriptive epidemiologic analysis provides foundational population-level assessment data to guide the development of culturally adapted, population-specific physical activity and mental health promotion strategies [50]. These findings establish an essential evidence foundation for informing evidence-based national health policies to address the persistent mental health burden in Korea’s post-pandemic context [51].

## Figures and Tables

**Table 1:** General characteristics of study participants from the 2023 Korea Community Health Survey (N = 228,249).

Variables			N (%)
Sex		Men	104,995 (46.0)
		Women	123,254 (54.0)
Age (years)		20–39	45,686 (20.0)
		40–59	76,175 (33.4)
		≥60	106,388 (46.6)
Marital status		Marriage	144,836 (63.5)
		Separation	6844 (3.0)
		Bereavement	26,306 (11.5)
		Divorce	12,198 (5.3)
		Single	38,065 (16.7)
Highest education level		High school graduate or less	136,308 (59.7)
		College graduate	82,317 (36.1)
		Graduate school or higher	9624 (4.2)
Physical Activity (per weeks)	Moderate	No activity	144,053 (63.1)
		1–2 days	27,414 (12.0)
		3–4 days	24,799 (10.9)
		5–7 days	31,983 (14.0)
	Vigorous	No activity	171,654 (75.2)
		1–2 days	24,272 (10.6)
		3–4 days	16,740 (7.3)
		5–7 days	15,583 (6.8)
Total			228,249 (100.0)

Note: Percentages may not sum to 100% due to rounding and a small proportion of missing responses in the KCHS dataset.

**Table 2:** Differences in depression symptoms according to general characteristics.

Variables		Little Interest In Work	Depression or Hopelessness	Sleep Disorder	Tiredness	Anorexia or Overeating	Feeling Bad About Yourself	Attention Deficit	Slow Movement or Speech	Suicidal Ideation
Sex	Men	1.31 ± 0.65	1.19 ± 0.50	1.41 ± 0.77	1.55 ± 0.75	1.21 ± 0.53	1.09 ± 0.37	1.08 ± 0.34	1.04 ± 0.27	1.06 ± 0.30
	Women	1.33 ± 0.66	1.30 ± 0.60	1.59 ± 0.89	1.70 ± 0.82	1.32 ± 0.65	1.11 ± 0.40	1.10 ± 0.39	1.05 ± 0.30	1.09 ± 0.37
	t	−7.091 ***	−45.710 ***	−52.608 ***	−45.518 ***	−45.041 ***	−10.171 ***	−17.720 ***	−8.973 ***	−22.630 ***
Age (years)	20–39 (a)	1.45 ± 0.75	1.24 ± 0.54	1.46 ± 0.79	1.71 ± 0.84	1.32 ± 0.64	1.11 ± 0.42	1.09 ± 0.37	1.04 ± 0.25	1.07 ± 0.32
	40–59 (b)	1.31 ± 0.62	1.23 ± 0.53	1.44 ± 0.78	1.63 ± 0.78	1.24 ± 0.55	1.10 ± 0.37	1.07 ± 0.32	1.03 ± 0.23	1.05 ± 0.29
	60 over (c)	1.28 ± 0.63	1.27 ± 0.59	1.57 ± 0.90	1.60 ± 0.79	1.28 ± 0.62	1.10 ± 0.39	1.10 ± 0.39	1.07 ± 0.34	1.10 ± 0.38
	*F*	1042.874 ***	175.427 ***	632.938 ***	333.308 ***	266.443 ***	33.722 ***	181.287 ***	352.879 ***	343.607 ***
	Post hoc	a > b > c	c > a > b	c > a > b	a > b > c	a > c > b	a > c > b	c > a > b	c > a > b	c > a > b
Marital status	Marriage (a)	1.28 ± 0.60	1.21 ± 0.51	1.46 ± 0.80	1.60 ± 0.77	1.23 ± 0.55	1.08 ± 0.33	1.07 ± 0.32	1.04 ± 0.24	1.05 ± 0.28
	Separation (b)	1.29 ± 0.62	1.27 ± 0.58	1.49 ± 0.83	1.58 ± 0.77	1.25 ± 0.57	1.12 ± 0.43	1.08 ± 0.34	1.04 ± 0.26	1.08 ± 0.35
	Bereavement (c)	1.35 ± 0.70	1.39 ± 0.68	1.72 ± 0.97	1.71 ± 0.85	1.41 ± 0.75	1.14 ± 0.45	1.16 ± 0.49	1.11 ± 0.43	1.16 ± 0.49
	Divorce (d)	1.42 ± 0.76	1.40 ± 0.72	1.69 ± 0.98	1.73 ± 0.88	1.35 ± 0.70	1.22 ± 0.58	1.12 ± 0.44	1.06 ± 0.37	1.15 ± 0.48
	Single (e)	1.46 ± 0.77	1.26 ± 0.57	1.48 ± 0.81	1.66 ± 0.82	1.30 ± 0.63	1.13 ± 0.44	1.10 ± 0.39	1.05 ± 0.29	1.08 ± 0.36
	*F*	674.184 ***	509.883 ***	744.060 ***	193.518 ***	582.692 ***	525.331 ***	395.964 ***	397.171 ***	727.552 ***
	Post hoc	e > d > c > a,b	c,d > b,e > a	c,d > b,e > a	c,d > e > a,b	c > d > e > a,b	d > c > b,e > a	c > d > e > a,b	c > d > a,b/e > a	c > d > b,e > a
Highest Education level	High school graduate or less (a)	1.31 ± 0.65	1.28 ± 0.59	1.56 ± 0.89	1.62 ± 0.80	1.28 ± 0.63	1.11 ± 0.41	1.10 ± 0.39	1.06 ± 0.33	1.09 ± 0.38
	College graduate (b)	1.35 ± 0.67	1.22 ± 0.51	1.43 ± 0.76	1.64 ± 0.78	1.26 ± 0.57	1.09 ± 0.36	1.07 ± 0.33	1.03 ± 0.22	1.05 ± 0.27
	Graduate school or higher(c)	1.26 ± 0.57	1.18 ± 0.47	1.39 ± 0.71	1.63 ± 0.76	1.23 ± 0.53	1.07 ± 0.32	1.07 ± 0.292	1.03 ± 0.19	1.04 ± 0.23
	*F*	154.473 ***	379.823 ***	766.210 ***	12.243 ***	79.609 ***	101.629 ***	147.186 ***	347.954 ***	521.760 ***
	Post hoc	b > a > c	a > b > c	a > b > c	b > a	a > b > c	a > b > c	a > b,c	a > b,c	a > b > c

Note: Mean ± Standard deviation; *** *p* < 0.001.

**Table 3:** Differences in MPA and VPA participation levels according to general characteristics.

Variables		MPA (Days/Week)	VPA (Days/Week)
Sex	Men	1.57 ± 2.28	1.04 ± 1.88
	Women	1.21 ± 2.06	0.59 ± 1.44
	*t*	39.112 ***	62.935 ***
Age (years)	20–39 (a)	1.50 ± 2.05	1.28 ± 1.90
	40–59 (b)	1.46 ± 2.14	0.94 ± 1.75
	60 over (c)	1.27 ± 2.23	0.48 ± 1.44
	*F*	267.797 ***	4229.019 ***
	Post hoc	a > b > c	a > b > c
Marital status	Marriage (a)	1.45 ± 2.23	0.79 ± 1.67
	Separation (b)	1.44 ± 2.22	0.81 ± 1.72
	Bereavement (c)	0.91 ± 1.96	0.26 ± 1.08
	Divorce (d)	1.18 ± 2.07	0.66 ± 0.57
	Single (e)	1.47 ± 2.06	1.25 ± 0.91
	*F*	396.730 ***	1406.548 ***
	Post hoc	a,b,e > d > c	e > a,b > d > c
Highest education level	High school graduate or less(a)	1.29 ± 2.22	0.58 ± 1.53
	College graduate (b)	1.50 ± 2.09	1.11 ± 1.82
	Graduate school or high (c)	1.58 ± 2.06	1.20 ± 1.82
	*F*	273.967 ***	2994.389 ***
	Post hoc	c > b > a	c > b > a

Note: Mean ± Standard deviation; MPA, moderate physical activity; VPA, vigorous physical activity; *** *p* < 0.001.

**Table 4:** Differences in depressive symptom scores according to levels of moderate and vigorous physical activity.

Variables		Little Interest in Work	Depression or Hopeless	Sleep Disorder	Tiredness	Anorexia or Overeating	Feeling Bad About Yourself	Attention Deficit	Slow Movement or Speech	Suicidal Ideation
MPA (per week)	No activity (a)	1.34 ± 0.68	1.27 ± 0.59	1.53 ± 0.86	1.64 ± 0.81	1.29 ± 0.63	1.11 ± 0.41	1.10 ± 0.39	1.06 ± 0.32	1.09 ± 0.37
	1–2 days (b)	1.34 ± 0.63	1.23 ± 0.51	1.47 ± 0.79	1.64 ± 0.76	1.27 ± 0.57	1.10 ± 0.37	1.09 ± 0.34	1.04 ± 0.24	1.06 ± 0.29
	3–4 days(c)	1.30 ± 0.61	1.20 ± 0.49	1.45 ± 0.78	1.58 ± 0.74	1.24 ± 0.55	1.08 ± 0.34	1.07 ± 0.32	1.03 ± 0.23	1.05 ± 0.28
	5–7 days (d)	1.28 ± 0.62	1.20 ± 0.51	1.45 ± 0.82	1.59 ± 0.79	1.22 ± 0.54	1.08 ± 0.35	1.07 ± 0.31	1.03 ± 0.22	1.06 ± 0.29
	*F*	83.725 ***	241.031 ***	144.947 ***	73.413 ***	141.034 ***	66.378 ***	97.800 ***	137.487 ***	165.625 ***
	Post hoc	a,b > c > d	a > b > c,d	a > b > c,d	a,b > c,d	a > b > c > d	a > b > c,d	a > b > c,d	a > b,c,d	a > b,c,d
VPA (per week)	No activity (a)	1.32 ± 0.66	1.27 ± 0.58	1.53 ± 0.86	1.63 ± 0.80	1.28 ± 0.62	1.11 ± 0.40	1.09 ± 0.38	1.05 ± 0.31	1.08 ± 0.36
	1–2 days (b)	1.35 ± 0.65	1.22 ± 0.50	1.45 ± 0.77	1.65 ± 0.76	1.27 ± 0.56	1.10 ± 0.37	1.08 ± 0.34	1.03 ± 0.22	1.05 ± 0.27
	3–4 days(c)	1.32 ± 0.63	1.20 ± 0.49	1.43 ± 0.75	1.59 ± 0.74	1.24 ± 0.54	1.09 ± 0.36	1.07 ± 0.33	1.03 ± 0.21	1.05 ± 0.27
	5–7 days (d)	1.31 ± 0.65	1.19 ± 0.50	1.43 ± 0.79	1.58 ± 0.80	1.22 ± 0.55	1.09 ± 0.37	1.07 ± 0.32	1.03 ± 0.23	1.06 ± 0.29
	*F*	17.377 ***	174.441 ***	159.705 ***	43.715 ***	65.220 ***	19.271 ***	37.143 ***	85.893 ***	113.813 ***
	Post hoc	b > a,c/b > d	a > b > c,d	a > b,c/a > d	b > a > c,d	a > b > c > d	a > b/a > c,d	a > b > c/a > d	a > b,c,d	a > b,c,d

Note: Values are mean ± SD. Differences generally ranged from 0.02 to 0.12 points (≈1–4% of the total scale range). While all comparisons were statistically significant (*** *p* < 0.001), effect sizes (η^2^ ≥ 0.06) indicate small individual-level differences but potentially meaningful population-level patterns.

## Data Availability

Publicly available datasets were analyzed in this study. These data can be found here: https://chs.kdca.go.kr/chs/rdr/rdrInfoProcessMain.do (accessed on 25 July 2025).
